# Balance Impairment in Radiation Induced Leukoencephalopathy Patients Is Coupled With Altered Visual Attention in Natural Tasks

**DOI:** 10.3389/fneur.2018.01185

**Published:** 2019-01-23

**Authors:** Ioannis Bargiotas, Albane Moreau, Alienor Vienne, Flavie Bompaire, Marie Baruteau, Marie de Laage, Matéo Campos, Dimitri Psimaras, Nicolas Vayatis, Christophe Labourdette, Pierre-Paul Vidal, Damien Ricard, Stéphane Buffat

**Affiliations:** ^1^UMR 8257 Cognition and Action Group (CNRS, Service de Santé des Armées, Université Paris Descartes Paris Sorbonne Cité), Paris, France; ^2^CMLA, ENS Cachan, CNRS, Université Paris-Saclay, Cachan, France; ^3^Service de neurologie, Hôpital d'Instruction des Armées Percy, Service de Santé des Armées, Clamart, France; ^4^OncoNeuroTox Center, Paris, France; ^5^School of Automation, Hangzhou Dianzi University, Zhejiang, China; ^6^Ecole du val de Grâce, Service de Santé des Armées, Paris, France; ^7^Institut de Recherche Biomédicale des Armées, Brétigny-sur-Orge, France

**Keywords:** balance control, attention, ecological tasks, eye movements, machine learning, radiation-induced leukoencephalopathy, dysexecutive syndrome

## Abstract

**Background:** Recent studies have shown that alterations in executive function and attention lead to balance control disturbances. One way of exploring the allocation of attention is to record eye movements. Most experimental data come from a free viewing of static scenes but additional information can be leveraged by recording eye movements during natural tasks. Here, we aimed to provide evidence of a correlation between impaired visual alteration in natural tasks and postural control in patients suffering from Radiation-Induced Leukoencephalopathy (RIL).

**Methods:** The study subjects were nine healthy controls and 10 patients who were diagnosed with RIL at an early stage, with isolated dysexecutive syndrome without clinically detectable gait or posture impairment. We performed a balance evaluation and eye movement recording during an ecological task (reading a recipe while cooking). We calculated a postural score and oculomotor parameters already proposed in the literature. We performed a variable selection using an out-of-bag random permutation and a random forest regression algorithm to find: (i) if visual parameters can predict postural deficit and, (ii) which are the most important of them in this prediction. Results were validated using the leave-one-out cross-validation procedure.

**Results:** Postural scores indeed were found significantly lower in patients with RIL than in healthy controls. Visual parameters were found able to predict the postural score of RIL patients with normalized root mean square error (RMSE) of 0.16. The present analysis showed that horizontal and vertical eye movements, as well as the average duration of the saccades and fixations influenced significantly the prediction of the postural score in RIL patients. While two patients with very low MATTIS-Attention sub score showed the lowest postural scores, no statistically significant relationship was found between the two outcomes.

**Conclusion:** These results highlight the significant relationship between the severity of balance deficits and the visual characteristics in RIL patients. It seems that increased balance impairment is coupled with a reduced focusing capacity in ecological tasks. Balance and eye movement recordings during a natural task could be a useful aspect of multidimensional scoring of the dysexecutive syndrome.

## Introduction

Radio-induced Leukoencephalopathy (RIL from now on) is a diffuse pathology of the white matter, consecutive to brain radiotherapy (RT) that was used to treat brain tumors. It is currently the most frequent and threatening delayed complication of cerebral RT. Symptoms may be manifested months or even decades after either cerebral RT alone or cerebral RT combined with chemotherapies (1). Cognitive impairment such as attention or memory deficits are the primary manifestations of the disease followed by balance and gait impairment and, at an advanced stage, urinary incontinence (1, 2). They have a significant impact on the patient's life, often permanently affecting his/her autonomy. Progressively, RIL patients may also suffer from severe dementia and total loss of autonomy (2–4). In the severe stage of RIL syndrome, patients may benefit from specific motor and cognitive re-education programs. Early diagnosis could be advantageous to prevent balance and gait disability.

Cognitive deficits are the earliest signs of RIL and affect mainly the attention and executive functions in a fronto-subcortical pattern with consequences on long-term memory and information processing (1, 3, 5–7). Anatomical white matter alterations after radiotherapy and/or chemotherapy have been recently correlated with cognitive impairment (8, 9). As dorsal periventricular tracts of the corona radiata -preferentially altered whatever the irradiation scheme- disrupt, patients progressively display balance and gait impairment resembling apraxia developing into dysexecutive syndrome (2). Chronic oculomotor dysfunction due to radiotherapy has been also previously reported. Clinical cases of patients with RIL reported deteriorated smooth pursuit eye movement with occasional saccadic intrusions as well as altered voluntary saccades. Still, the mechanisms remain unclear. Eye movement recordings have been reported as reflections of tenuous cognitive deficits before their clinical manifestation (10). Oculomotor and balance functions are also closely interwoven. Defective gaze behavior has been associated with impaired posture control in elderly (11) as well as in Parkinsonian patients (12) and it has been proposed as a biomarker of impaired posture (13). Several studies explored the interrelation between eye-movements and posture, since several brain regions (parietotemporal cortex, brainstem, superior colliculus, and cerebellum) are involved in both eye movements and postural control (14, 15).

Attention is necessary to both postural control (16) and eye movements (17, 18). The frontal cortex which is strongly connected to the parietal areas (19), may also play an important role in the interaction between visual and postural systems (20). Ecological tasks enable researchers to study the executive control of gaze and have been used in several contexts in recent years (14). In this context, it has been highlighted how prominent the role of attention and task demand toward explaining oculomotor behavior can be.

The newly proposed data mining techniques have been shown to have an added value to the exploitation of the available datasets, especially when multiple variables occur and the number of available individuals is limited. In this study, we explored posture and oculomotor control in patients recently diagnosed with RIL (using brain MRI), at the early stages of dysexecutive syndrome. At the process of the patients' neurological examination, no balance or gait impairment was detected. The present work, attempts to investigate (a) the level of association between early balance/gait and oculomotor deficits in RIL patients and (b) whether these balance/gait deficits reflect patients' cognition impairment. Early detection of balance, gait and oculomotor abnormalities in RIL patients could lead to new rehabilitation strategies and reassessment of current therapeutic interventions.

## Material and Methods

### Participants

Ten patients between 19 and 63 years old (average age 50.9 ± 15.9 years old; 4 women) and nine healthy participants between 27 and 54 years old (mean age 43.4 ± 10.2 years old; 3 women) participated in this study. The patients were enrolled in the Neurology department at Percy Hospital, Clamart (France), referred for RIL after a brain tumor treatment or metastatic prophylactic cerebral irradiation. Patients were examined by neurologists of our center, and inclusion criteria were: (i) Patients diagnosed with RIL according to MRI and clinical criteria as previously detailed (2). All patients had extensive FLAIR hyperintensities also in other lobes (at least one), different than this of the initial tumor location, as well as in corona radiata. (ii) with dysexecutive syndrome as defined by the GREFFEX criteria on the cognitive battery (2), (iii) with no complains of balance or gait impairment, (iv) normal visual acuity (corrected visual acuity with glasses was permitted) (v) having understood and agreed on the aim of the study and given informed consent. Exclusion criteria were (i) vestibular or proprioceptive dysfunctions according to the neurologist examination, (ii) balance impairment detected at the visual Romberg test performed by the neurologist, (iii) ongoing psychiatric pathology, (iv) unable to understand and follow instructions.

The control subjects were all naïve regarding the aim of the study. They were recruited among the hospital personal. They had no complaint and no history of brain radiation, traumatic brain injury. Table 1 presents a synthetic view of characteristics for the participants' sample.

**Table 1:** Synthetic view of characteristics for the participants' sample.

**Participant characteristics**	**Patients Mean (±*SD*)**	**Control subjects Mean (±*SD*)**
Mean age (in years)	50.9 ±15.9	43.4 ±10.2
Women	4	3
Delay since brain irradiation (years)	13.6 ± 13	–
**TYPE OF TUMOR**		
Glioma	5	–
Medulloblastoma	2	–
Astrocytoma	1	–
Prophylactic brain irradiation	1	–
Primitive central nervous system lymphoma	1	–

### Ethical Statement

This study was registered at ethical committee CPP Nord Ouest III with the number ID RCB: 2017-A01538-45. All participants (controls and patients) received written and oral information and gave written consent.

### Ecological Tasks Assessment

**Figure 1:**
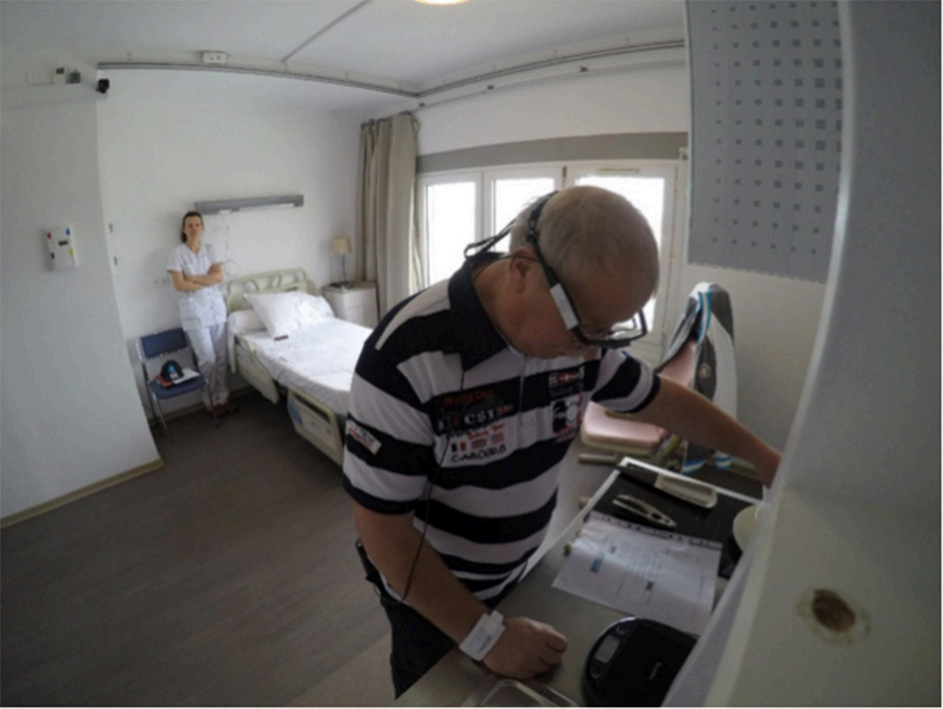
Snapshot of a patient while reading the recipe. Note that the participant is wearing a Tobii glasses 2® eye tracking device and that there is a Clinical Research Nurse in the background. Written informed consent was obtained from the participant for the publication of this image.

The participants were instructed to follow a recipe to prepare a chocolate cake (See Table A1). During the entire task, a nurse accompanied both patients and control. The participants were equipped with a mobile eye tracking system (Tobii Pro Glasses 2, coupled with the Tobii pro lab analyzer edition software, with a sampling frequency of 100 Hz). A calibration of the eye tracker was made at the beginning of the experiment. Figure 1 provides a snapshot of the “reading recipe while cooking” task.

### Data Analysis

For the purpose of this study, we concentrated the oculomotor analysis on the time periods when the participants read the recipe. Algorithms and statistical analysis has been performed in Matlab platform R2018a.

#### Oculomotor Parameters

Most of the calculated parameters have been previously proposed as visual characteristic (21). The main idea of this feature engineering process is to base our analysis to already known parameters from the oculomotor scientific community to facilitate the reader's interpretation. However, in order to further exploit the richness of the eye movement in time, characteristics inspired by analytical studies with similar two-dimensional datasets (such as the center of mass coordinates changes in postural control research) (22, 23) were applied in the eye movement datasets. Table 2 provides the names and the description/values (where needed) of the biomarkers that were initially included in the model.

**Table 2:** Visual parameters that were initially calculated and included in the model.

**Biomarkers**	**Description**
**DYNAMIC VISUAL PARAMETERS**	
RangeX (degrees/s)	Range of horizontal eye movement per second during task
RangeY (degrees/s)	Range of vertical eye movement per second during task
RatioRange	RatioX/RatioY
VarianceX (degrees/s)	Variance of horizontal eye movement per second during task
VarianceY (degrees/s)	Variance of vertical eye movement per second during task
VelocityX (degrees/s)	Average instant velocity of horizontal eye movement during task
VelocityY (degrees/s)	Average instant velocity of vertical eye movement during task
Velocity (degrees/s)	Average instant velocity of eye movement during task
EllArea(degrees/s)Horizontal and vertical field of view	Confidence ellipse that covers the 95% of the trajectory points. The horizontal and vertical field of view per second are the axes of the ellipse.
**STANDARD VISUAL PARAMETERS**	
MeanFix (ms)	Average duration of fixations during task
VarianceFix (ms)	Variance of durations of fixations during task
SkewFix	Skewness of durations of fixations during task
KurtFix	Kurtosis of durations of fixations during task
MeanSac (ms)	Average duration of saccades during task
VarianceSac (ms)	Variance of durations of saccades during task
SkewSac	Skewness of durations of saccades during task
KurtSac	Kurtosis of durations of saccades during task
Fix2SacNratio	Number of Fixations/Number of Saccades

*This table separates the parameters into dynamic [mostly inspired by the posture evaluation literature (22, 23) (upper part), and more standard visual parameters (Lower part)]*.

#### Statistical Analysis

##### Postural differences between RIL patients and controls

All individuals completed the basic Romberg test (upright position, without shoes, feet placed in comfort for the patient but in the shoulders' projection area on the force platform, arms laying at the side, 25 s eyes open, 25 s closed eyes) on the Wii balance board^®^ (WiiBB). WiiBB has non-constant frequency during the record and so the signal was resampled at 25 Hz using the SWARII algorithms previously described (24). For acclimatization purposes with WiiBB, a period of 35 s (minimum) has been kept before the open and closed-eyes recordings. Statokinesigrams were analyzed using the LAGMM (Local Analysis of Statokinesigrams using Gaussian Mixture Models) algorithm already proposed in (22) for statokinesigram datasets and it is available online (http://taureau.pppcmla.ens-cachan.fr/). Briefly, the proposed model creates a multidimensional profile for every individual using both open and closed eyes parameters and analyses their center of pressure (CoP) trajectories in “local parts” (time frames). The scores per individual are initially given by the value 1 minus the percentage of unquiet periods for both eyes closed and eyes open. The final score is given by the average of these two scores and it is scaled to the 0–100 scale. The given scores (0: Bad, 100: Excellent) for every individual were analyzed using the univariate non-parametric Wilcoxon test in order to see if there is a significant difference between controls and RIL patients.

##### Oculomotor and posture control correlation in RIL patients

Our objective was to propose a model that finds significant elements of ocular-postural coupling particularly for the RIL patients. Therefore, we checked the power of visual characteristic to predict the postural score only in RIL patients. We performed a regression prediction using the random forests algorithm (25) only for the RIL patients. Briefly, random forest uses multiple weak classifiers (such as decision trees) using random subsamples (randomly selected observations and biomarkers (*i*) for every tree) of the initial training sample and merges their results in order to get the final classification result. Due to the limited available dataset, results were validated using the well-known leave-one-out validation. Dataset was split N times where train-test was the N-1 and test set was every single individual one.

Moreover, in order to evaluate the influence of every variable in predicting the right label, we estimated the well-known out-of-bag predictor importance by random permutation (26). Briefly, the more critical is the predictor, the more important would be the affectation of the model error (d). The permutation of a non-influential predictor will have minimum or no effect on the model's error. So the final importance is given by

(1) $$\begin{array}{r} Imp_{i}={\bar{d}}_{i}/\sigma_{i} \end{array}$$

Where *Imp*_*i*_ the importance of every biomarker *i*, ${\bar{d}}_{i}$ the average change of error after random permutation of biomarker *i* from trees that *i* was selected and σ_*i*_ is the standard deviation of the *d*_*i*_for trees that *i* was selected. The out-of-bag predictor importance was run five times. Variables that the 25% quartile of Imp was >0.1 were marked as significant variables.

#### MATTIS-Attention Subscore and Correlation With Posture

As mentioned previously, all included patients had impaired scores on the tests from the GREFEX battery in a manner that dysexecutive syndrome could be diagnosed according to the GREFFEX criteria (27). However, it was difficult to give a general score of their cognitive impairment that takes into account all the scores of the battery. We assumed that the attention subscore of the MATTIS scale, included in the patients' GREFFEX battery (27), reflects their global cognitive impairment. Table 3 summarizes the patients' MATTIS-Attention subscores. Below 31/37, the score is considered pathological (28).

**Table 3:** Patients' MATTIS-Attention sub-scores.

**Patient**	**MATTIS attention sub-score**
Patient 1	23/37
Patient 2	25/37
Patient 3	34/37
Patient 4	34/37
Patient 5	34/37
Patient 6	35/37
Patient 7	36/37
Patient 8	36/37
Patient 9	37/37
Patient10	37/37

Moreover, we used linear Pearson correlation for MATTIS-Attention and postural score in order to check the association of the posture dysfunction with the intensity of the cognitive impairment.

## Results

### Postural Differences Between RIL Patients and Controls

**Figure 2:**
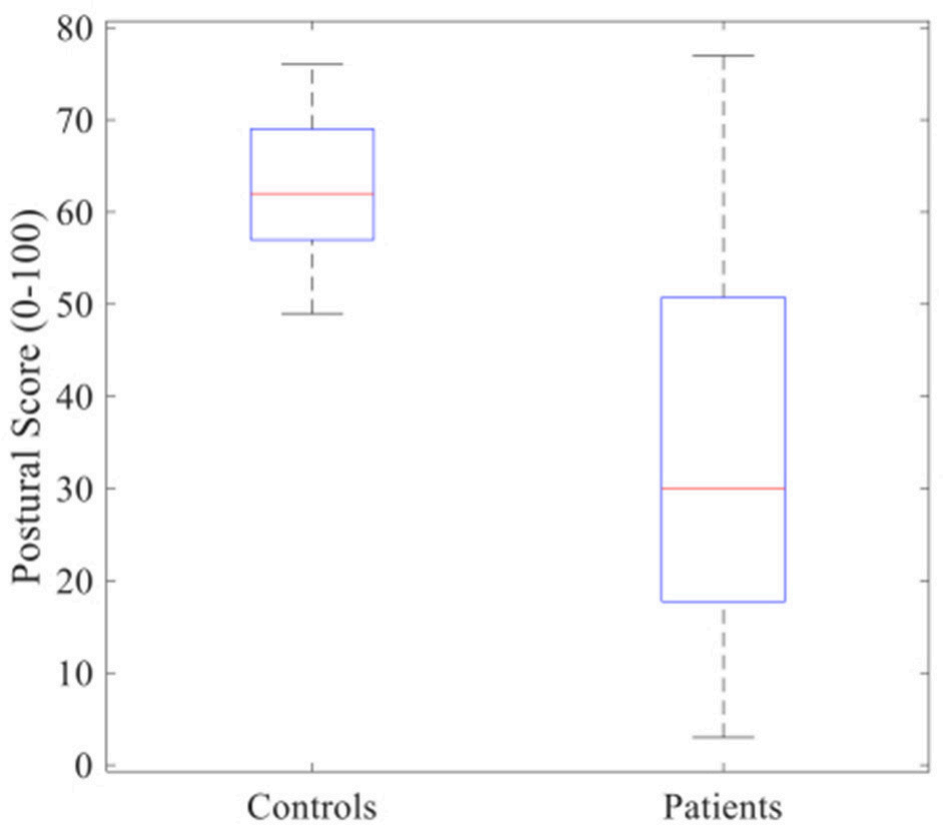
Boxplot of postural score between controls and patients who suffer from radio-induced leukoencephalopathy (RIL). Red lines inside the boxes indicate median and end of boxes indicate the whiskers. Non-parametric Wilcoxon test showed that RIL's score was found significantly lower compared to those of healthy controls (*p* < 0.01).

Postural control was found significantly lower in patients [30 (17, 50)] [median, (whiskers)] than in controls individuals [62 (57, 69)] (*p* < 0.01). The boxplot in Figure 2 below shows the clear separation between the two groups.

### Visual Parameters: Differences Between RIL Patients and Controls

Table 4 below summarizes the values of the calculated parameters.

**Table 4:** Average (±*SD*) of the included variables for RIL patients with relatively low and medium postural score.

**Biomarkers**	**RIL Posture score < 30 (*N* = 5)**	**RIL Posture score>30 (*N* = 5)**	**Controls (all have Posture score>30)**
**DYNAMIC VISUAL PARAMETERS**			
RangeX (degrees/s)	14.6 ± 5.6	6.0 ± 1.6	10.7 ± 4.5
RangeY (degrees/s)	9.5 ± 3.7	3.4 ± 1.0	5.5 ± 2.1
RatioRange	1.6 ± 0.6	2.0 ± 0.8	2.1 ± 0.8
VarianceX (degrees/s)	4.8 ± 1.7	2.1 ± 0.5	3.6 ± 1.4
VarianceY (degrees/s)	3.1 ± 1.1	1.2 ± 0.4	1.8 ± 0.7
VelocityX (degrees/s)	65 ± 22	23 ± 12	67 ± 87
VelocityY (degrees/s)	52 ± 8	18 ± 8	38 ± 31
Velocity (degrees/s)	92 ± 91	33 ± 15	85 ± 97
EllArea(degrees/s), (field of view) (Horizontal/Vertical)	H:27.6 ± 9, V:10 ± 3.4	H:12 ± 2.5, V:3.7 ± 1.2	H:20.5 ± 7, V:6.4 ± 2.7
**STANDARD VISUAL PARAMETERS**			
MeanFix (ms)	153 ± 31	316 ± 141	226 ± 89
VarianceFix (ms)	169 ± 63	318 ± 149	134 ± 60
SkewFix	6.1 ± 4.2	3.1 ± 1.6	1.7 ± 0.8
MeanSac (ms)	52 ± 11	41 ± 5	44 ± 9
VarianceSac (ms)	39 ± 28	28 ± 11	36 ± 14
SkewSac	3.2 ± 1.8	1.8 ± 0.7	2.7 ± 1.6
Fix2SacNratio	0.37 ± 0.15	1.05 ± 0.36	0.74 ± 0.35

*RIL patients with relatively higher postural score had many ocular parameters significantly different (see Table 5 below). Controls were presented in order to help the comparison between these populations. Worth to be noted that sometimes controls present values closer to the RIL with low postural control than those with relatively higher postural score*.

### Visual Parameters and Posture in RIL Patients

**Figure 3:**
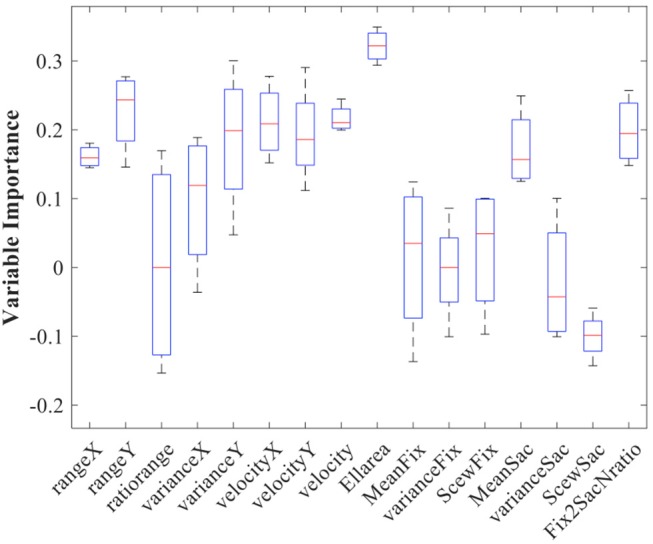
Oculomotor Parameters' importance resulted by the prediction importance algorithm. Red lines indicate the median and the horizontal lines of boxes indicate the whiskers. The biomarkers with low whisker >0.1 were considered as predictors that have very high possibility to have a beneficial effect on the final predictions.

Considering only the RIL population, we first checked the importance of the calculated parameters. Figure 3 shows the relative parameters' importance, which allows us to predict the final postural score of every participant.

The variables that have been finally selected are presented in Table 5.

**Table 5:** Biomarkers that were found valuable in the prediction process of the postural score for RIL patients.

**Biomarkers**	**Median (whiskers) of Importance**
**DYNAMIC VISUAL PARAMETERS**	
RangeX	0.16 (0.15, 0.18)
RangeY	0.24 (0.18, 0.27)
VarianceX	0.12 (0.02, 0.18)
VarianceY	0.19 (0.11, 0.26)
VelocityX	0.21 (0.17, 0.24)
VelocityY	0.18 (0.15, 0.23)
Velocity	0.21 (0.20, 0.22)
EllArea	0.32 (0.29, 0.34)
**STANDARD VISUAL PARAMETERS**	
MeanSac	0.15 (0.12, 0.21)
Fix2SacNratio	0.18 (0.15, 0.23)

*Horizontal and vertical eye movement increase significantly with the loss of postural control (see Table 4 above) and also the saccades become numerous and more extensive (in duration)*.

Some of the selected parameters in Table 5 (see also Tables 3, 4) might have a certain overlap in terms of explanatory power (ex. RangeY, VarianceY, VelocityY). These variables should be seen more as unity (as a profile) rather than three independent and different parameters. Therefore, we generally observed that movement in horizontal (RangeX, VarianceX, VelocityX) and vertical axes (ex. RangeY, VarianceY, VelocityY) are both increased significantly with the degradation of postural control (Table 4). Considering the standard oculomotor parameters, the average duration of the saccades was also increased and the average fixation period has been dramatically dropped (see Table 4). Interestingly, not only durations but also the ratio between numbers of fixations and the number of saccades per second was decreased with the decrease in postural score.

**Figure 4:**
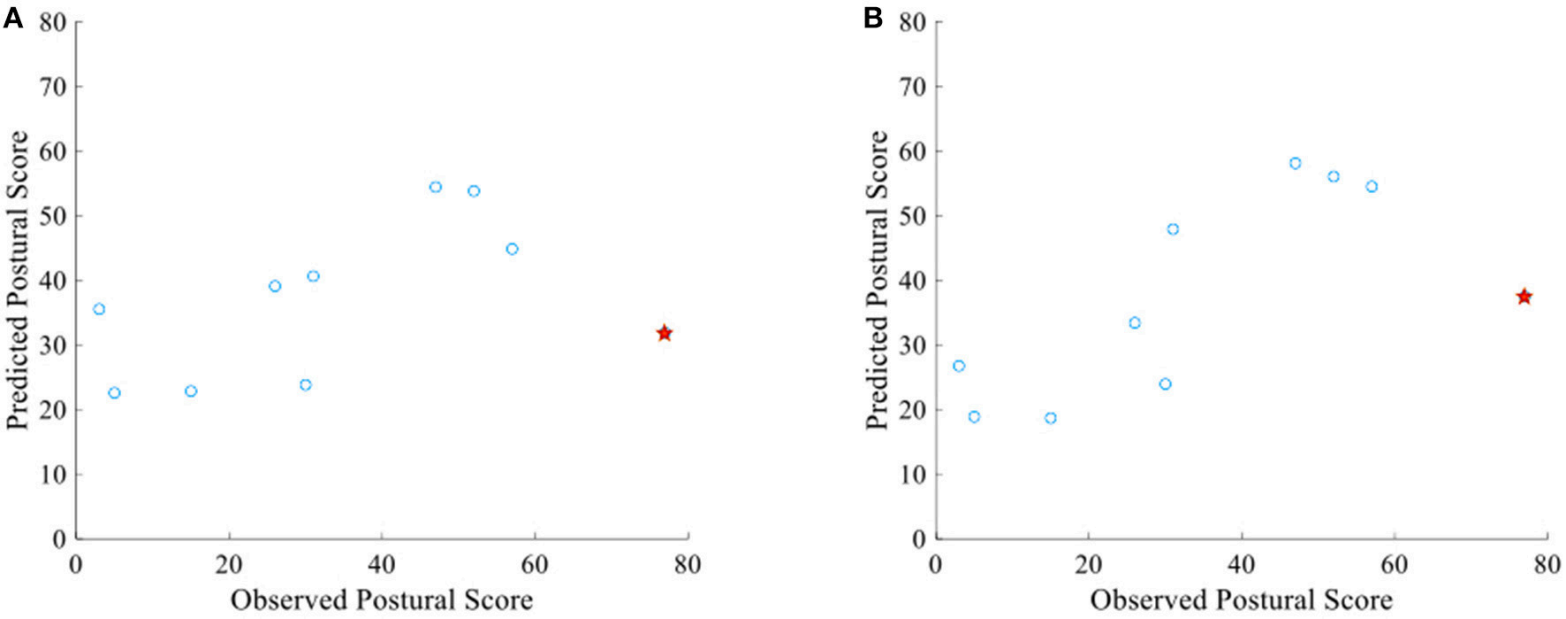
**(A)** Scatter plot between observed and predicted postural score of RIL patients using all the oculomotor biomarkers mentioned in Table 2 (RMSE = 0.2). **(B)** Scatter plot between observed and predicted postural score using only the selected oculomotor biomarkers mentioned in Table 5 (RMSE = 0.16). Both graphs contain an outlier prediction which increases the RMSE significantly (Red star).

Figure 4A below shows the scatter plot between the observed postural score and the predicted one (by oculomotor parameters). The prediction is fairly accurate with an RMSE = 0.2 (Normalized value–RMSE divided by 100–0). However, this value is increased by an individual (red STAR in the graph) that its prediction was not accurate. The RMSE without the outlier drops to 0.15. On the other hand, when we re-run the model after the inclusion of only the important parameters (Table 5), the prediction accuracy has been increased (RMSE = 0.16) (Figure 4B). However, the same patient as previously has been relatively mispredicted. The RMSE decreased at 0.11 after the exclusion of this case.

### Posture and Attention

We correlated the measured postural score with the attention scale of the MATTIS-Attention subscore using the Pearson linear correlation coefficient. Despite the fact that the patients with lower MATTIS-Attention subscore also had a low postural score, (patients with 23/37 and 25/37 had a postural score of 5/100 and 26/100 respectively), all the other patients had score close to 37 and so we did not find any statistical significance in order to make safer conclusions (*r* = 0.39, *p* = 0.28).

## Discussion

One should bear in mind that the neurological examination of patients in question who suffered from RIL at the early stage of dysexecutive function, did not show any balance or gait impairment. The objective of the present work was: (1) to initially explore possible postural control degradation in the patients involved using a simple Romberg test and data mining techniques and consequently associate this degradation with cognitive impairment, (2) to investigate whether there is any relation between the aforementioned postural deficits and the oculomotor control. It was shown that the horizontal and the vertical eye movements as well as the average duration of the saccades are significantly increased in RIL patients with relatively low postural control compared to those with higher postural control. What's more, critical attentional deficits seemed to be coupled with postural impairment leading us to believe that further research into a larger population is required to validate these preliminary findings.

### Postural and Visual Deficits in RIL Syndrome

This study is the first detailed report to our knowledge which connects the postural and visual deficits in a RIL patient's cohort. Despite the fact that initially the patients were not diagnosed either with balance or with gait dysfunction caused by the treated tumor or the RIL, these results present scores indicating postural impairments or at least lower scores compared to the healthy controls. Besides, RIL patients were found to suffer from various visual impairments, which, in result, may have contributed to imperfect information processing during complex tasks, such as the ecological reading task assigned. This is of significant importance because usual clinical tests appear less sensitive toward detecting such issues, despite relevant patient complaints.

### Vision and Posture Association in RIL Patients

The proposed method offered the advantage of checking all parameters simultaneously and thus avoiding the consecutive univariate parametric or non-parametric tests (such as *T*-Tests or Wilcoxon with or without corrections) often criticized especially in exploratory studies (29). Although a predictive model has been used, this result should not be mistaken for a unidirectional causal relationship between oculomotor deficits and postural control. Our results should be seen mostly as a confirmation that vision deficits may reflect posture ones, and vice versa. The results seem promising in the sense that they strongly encourage further research in this direction, in order to gain a better insight into the neurological basis of the radiation-induced brain damage. Patients were characterized by an heterogeneity of the brain lesions caused by the tumor and a relative homogeneity of the delayed periventricular tracts disruption caused by brain irradiation. This taken into account, it is interesting to see that all patients showed both postural and visual impairments, even at various degrees. To our knowledge, such a phenomenon has never been reported in other conditions close to the radio induced leukoencephalopathy. However, recent studies have shown that there is a relation between saccades and posture in control populations. Saccadic eye movements affect posture by decreasing the magnitude of body sway both in children (30) and older adults (31). Three different mechanisms have been suggested, that work toward the visual stabilization of posture.

1. The afferent motion perception, which uses information contained in the optic flow to minimize retinal slip and stabilize the distance between the eye and visual scene.
2. The efferent motion perception, which is based on either the copy of motor command or extra visual muscle afferents that are consecutive to eye movements.
3. The attentional aspects that relate to the execution of the eye-movement task and possibly influence patients ‘postural performance in the present study.

Specifically, attentional demands involved during the reading task, are consistent to an adaptive resource sharing model (32), which postulates that postural and supra-postural tasks compete for the same limited attentional resources. The increased body sway in RIL patients, despite increased saccadic movements, suggests at least a disruption in the normal relationship between saccades and posture. The fact that the patients in the present study suffered from a cognitive impairment, without clinical evidence for postural deficit, presents some interesting caveats to the cognitive penetrability of posture. We may suppose that altered visual strategy has a detrimental effect on posture (33). Also, ignoring irrelevant visual information is paramount to attend and interpret the essential parts of a visual event. We can assume that the modifications found in RIL patients have a detrimental effect on the quality of the visual input, but also partly upset the attentional system, as happens in Parkinsonians (34). This explains why, until recently, balance control was described as a predominantly automated motor process, requiring almost no cognitive input. However, recent studies have shown that alterations in executive function and attention lead to balance control disturbances (35). Our hypothesis and results are in line with these recent findings.

### Limitations

There are several limitations we would like to address. In terms of chosen analysis, the current analysis (predictive model) highlighted the ability of oculomotor parameters to predict postural control, without excluding that posture parameters might also predict oculomotor deficits. Therefore, our results indicate a strong interrelation rather than a causal relationship between oculomotor deficits and postural control.

The limited number of available patients, especially in a single clinical center, restrains the evaluation. The fact that we could conduct such an experiment in the neurology department is a strong opportunity to better understand the RIL syndrome. Plans have been made to work toward a more multi-centric approach in the future. The fact that we did not find statistically significant correlation between postural score and MATTIS-Attention sub-score might be also due to the aforementioned lack of large cohort. However, it should be also mentioned that postural score derived by the basic Romberg test, which is not extremely demanding in terms of cognition, might be insufficient to reflect mild cognitive deficits. Richer Romberg protocols (such as dual task (36) which presumes that cognitive functions and postural control compete for limited attentional capacity (32, 37), might be more appropriate in order to acquire more sensitive postural scores.

Additionally, there is a delicate tradeoff between controllability and practical choices in an ecological setting. More precisely, it is necessary to use a cooking recipe and test the reading parameters for this task. The text has a procedural organization, and the corpus is related to a specific semantic field. A further point of contention is the age difference between patients and controls. Still, the primary visual issue between 40 and 50 years, namely presbyopia, cannot affect our results. To our knowledge, no difference has been reported in the statokinesigrams between 40 and 70 years old, either. Most changes in saccadic eye movement in healthy subjects occur after 60 years (38) and thus any age bias is expected to be minor.

### Conclusion

The proposed method was based on multi-dimensional machine learning techniques. It offered the advantage of checking the importance of the ocular parameters to explain the postural impairment in RIL patients, while avoiding the consecutive Wilcoxon tests often criticized in exploration studies. The results of the present study are as follows. (1) Most RIL patients have significantly lower postural control scores when compared to the healthy controls. (2) The severity of these postural deficits is strongly associated with the increased vertical and horizontal eye movements as well as with longer saccades. (3) No statistically significant association was found between postural score and MATTIS-Attention sub-score in RIL patients. However, the fact that two RIL patients with very low MATTIS-Attention sub-score showed very low postural score too, is an element that needs further investigation. A larger sample of participants suffering from a wider range of postural stability and cognitive deficits as well as a richer Romberg protocol might be required to emphasize the reliability of the present result. Additional measures will enable researchers to clarify the underlying nature of the neurological lesions that cause cognitive impairment. Future works should focus on correlations of the above postural and visual deficits with brain imaging (MRI) as well as on the increase (as possible) of the sample.

A further establishment of the present results in the future, would render the ecological protocols and parameters we propose, as a complement of the cognitive tests, a major assistance in assessing the stage of the patients' conditions and facilitating the patients' follow-up examinations. Such approaches may also have a positive effect on the rehabilitation strategies at an early RIL stage.

## Author Contributions

SB and DR: Conceived and designed the experiment; AM, AV, and MC: Recordings of participants; IB, SB, DR, and AV: Analyzed the data; IB, AV, AM, FB, DP, MdL, MB, and CL: Contributed reagents, material, analysis tools; IB, SB, AV, and DR: Wrote the article; IB, SB, DR, NV, and P-PV: Review the article.

### Conflict of Interest Statement

The authors declare that the research was conducted in the absence of any commercial or financial relationships that could be construed as a potential conflict of interest.

## Appendix

**Table A1:** Recipe and associated elements per instruction.

**Instruction**	**Elements/ actions**
1. Melt the chocolate and the butter in the microwave. Mix well the two ingredients (The microwave is already programmed, just press to start it).	• Chocolate bar Butter (opening and closing fridge) Container: choice Spoon: any choice Oven (opening + start + closing)
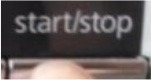
2. Mix the flour, the sugar and the egg yolks in a bowl.	• Flour Sugar Balance or measuring cup Eggs (opening and closing fridge) Containers (x2: white mixing and separation)
3. Stir the chocolate-butter mixture into the previous mixture.	• Optional: opening / closing oven if the mixture has not already been removed Spoon: choice Optional: dishwasher
4. Beat the egg whites with the electric mixer until stiff and fluffy.	• Electric mixer (plug, turn on) Whisks (to be installed on the mixer) Salad bowl where are the whites
5. Gently incorporate the egg whites in the mixture with a wooden spoon.	• Wooden spoon Salad bowl (egg whites, mix)
6. Pour the mixture into the mold.	• Bowl with mix Mold Spoon: choice

that actual recipe was presented in a more readable format, with a size font suitable for a distant reading.
